# *Caenorhabditis elegans* Predation on *Bacillus anthracis*: Decontamination of Spore Contaminated Soil with Germinants and Nematodes

**DOI:** 10.3389/fmicb.2017.02601

**Published:** 2018-01-05

**Authors:** Bettina Schelkle, Young Choi, Leslie W. Baillie, William Richter, Fatih Buyuk, Elif Celik, Morgan Wendling, Mitat Sahin, Theresa Gallagher

**Affiliations:** ^1^School of Pharmacy and Pharmaceutical Sciences, Cardiff University, Cardiff, United Kingdom; ^2^Battelle Biomedical Research Center, Columbus, OH, United States; ^3^Faculty of Veterinary Medicine, Department of Microbiology, University of Kafkas, Kars, Turkey; ^4^Avila Scientific, Christiansburg, VA, United States

**Keywords:** anthrax, remediation, environmentally friendly, *Caenorhabditis elegans* N2, L-alanine, inosine

## Abstract

Remediation of *Bacillus anthracis*-contaminated soil is challenging and approaches to reduce overall spore levels in environmentally contaminated soil or after intentional release of the infectious disease agent in a safe, low-cost manner are needed. *B. anthracis* spores are highly resistant to biocides, but once germinated they become susceptible to traditional biocides or potentially even natural predators such as nematodes in the soil environment. Here, we describe a two-step approach to reducing *B. anthracis* spore load in soil during laboratory trials, whereby germinants and *Caenorhabditis elegans* nematodes are applied concurrently. While the application of germinants reduced *B. anthracis* spore load by up to four logs depending on soil type, the addition of nematodes achieved a further log reduction in spore count. These laboratory based results suggest that the combined use of nematodes and germinants could represent a promising approach for the remediation of *B. anthracis* spore contaminated soil.

**Originality-Significance Statement:** This study demonstrates for the first time the successful use of environmentally friendly decontamination methods to inactivate *Bacillus anthracis* spores in soil using natural predators of the bacterium, nematode worms.

## Introduction

*Bacillus anthracis* is the causative agent of anthrax and in areas where the dormant spore form of the bacterium has contaminated the soil it represents an ever present threat to herbivores of economic and conservation importance (Hugh-Jones and de Vos, 2002; Knutsson et al., 2012; Salb et al., 2014). In the event of intentional spore release, large geographical areas can be rendered uninhabitable for extended periods of time (Manchee et al., 1994). *B. anthracis* spore viability in soil is stable over long periods of time (Wood et al., 2015). Hence, the ability to reduce the overall *B. anthracis* spore load in contaminated soil to a level that does not pose a threat to grazing animals or to human health using an environmentally friendly approach that maintains the integrity of the ecosystem is highly desirable (Raber et al., 2001, 2004; Sharp and Roberts, 2006; Pottage et al., 2014).

Remediation of *B. anthracis*-contaminated soil is challenging due to the ability of the pathogen to form spores (Driks, 2009); the concentrations of biocides such as formaldehyde or hypochlorite solutions required to inactivate spores are highly damaging to the environment (Manchee et al., 1994) and human health. However, in its vegetative form, the bacterium is considerably more sensitive to biocides and therefore a two-stage decontamination strategy may be more effective. In this approach, spores are treated with germinants, chemicals (L-alanine and inosine) that induce the spore to break down its protective shell even in the absence of favorable replication conditions, to convert the bacteria to the vegetative state that can then be treated with biocides (Omotade et al., 2014; Celebi et al., 2016). Indeed, germinants alone may be sufficient to reduce spore numbers in certain soil types (Bishop, 2014). The mechanisms behind this reduction are unclear but may relate to inhibition of full spore germination and/or the presence of natural predators in the soil such as bacteriophages, protozoa, and nematodes (Klobutcher et al., 2006; Dey et al., 2012; Rønn et al., 2012). Indeed increasing the ratio of predators to *B. anthracis* in the soil in combination with application of germinants has the potential to reduce soil contamination with minimum damage to the environment.

Free living nematodes are ubiquitous in soil, require a thin film of water to survive and are known to feed on bacteria, fungi, and protozoa (Neher, 2010); hence, their addition to *B. anthracis* contaminated soil for remediation efforts beyond the efficacy of germinants alone would not perturb the ecosystem, particularly if a locally isolated species was used. These and other characteristics, such as their extensive use as pesticides, make these soil predators ideal for use in a two-step approach to reducing the *B. anthracis* spore load in soil (Grewal et al., 2005).

This study aimed to determine whether germinants and the nematode *Caenorhabditis elegans* N2, when used in combination, could decrease overall *B. anthracis* burden in soil. *C. elegans* N2 is a well-established laboratory strain whose ease of maintenance and short propagation time in the laboratory make it an ideal model for the study (Stiernagle, 2006). Further, the nematode is known to feed non-selectively on a wide range of microbes in the wild and is largely associated with rotten fruit, not soil (Felix and Braendle, 2010; Felix and Duveau, 2012). In the context of reducing *B. anthracis* spores in the soil, the introduction of *C. elegans* N2 into soil has the benefit that the nematode is unlikely to survive beyond a few days once the food source, i.e., germinated *Bacillus* spp., is diminished so preventing the long term perturbation of the ecosystem (van Voorhies et al., 2005).

Laboratory studies have shown that *C. elegans* N2 can survive and propagate on fully virulent and attenuated variants of *B. anthracis*, including the Sterne 34F2 strain (Schelkle, personal observations); hence, the nematodes use the bacteria as a food source without any obvious detrimental effects. Bacterial fate was confirmed through experiments utilizing a green fluorescent protein (GFP)-tagged *B. anthracis* Sterne 34F2 strain. Microcosm studies to assess *B. anthracis* recovery from sterilized and unsterilized soil, as well as to investigate the effect of germinants on overall cultivable microorganisms were conducted. Finally, additional microcosm experiments to assess the feasibility of a combined germinant and nematode approach were carried out. For the microcosm studies, unsterilized soil from endemic anthrax-positive animal burial grounds in northeast Turkey, and both sterilized and not-sterilized soil from non-endemic (South East Wales) regions were used. For all experiments, bacteria were enumerated on *B. anthracis* selective polymyxin, lysozyme, ethylenediamine-tetraacetic acid, thallium acetate (PLET) and tryptic soy agar (TSA) media to determine if there was any advantage in using PLET over TSA as indicated by previous studies (Sjøstedt et al., 1997; Dragon and Rennie, 2001).

## Materials and Methods

All work using samples suspected of containing fully virulent *B. anthracis* was undertaken in accordance with the biosafety regulations of the Bio-Safety Level-3 laboratory set up of Kafkas University, Turkey, which is governed by a number of Turkish National regulations (Supplementary Material).

### Bacterial and *C. elegans* N2 Strain Propagation

*C. elegans* N2 was purchased from the Caenorhabditis Genetics Center (University of Minnesota, Minneapolis, MN, United States), propagated for two generations using standard culture methods, and then kept frozen in liquid nitrogen (-196°C) until required (Stiernagle, 2006). In preparation, aliquots of the frozen population were thawed and propagated for another two generations on *Escherichia coli* OP50 (obtained from the Caenorhabditis Genetics Center, University of Minnesota, Minneapolis, MN, United States) for use as a heterogeneous culture. Nematodes were removed from Nematode Growth Medium (NGM) with a 5 mL stream of M9 solution (Stiernagle, 2006) and gentle scraping with a spreader of the agar surface. The runoff containing *C. elegans* N2 was collected in a sterile 50 mL conical centrifuge tube (Falcon, Fisher Scientific, United Kingdom) and the process of adding M9 solution and gentle scraping of the surface of the NGM was repeated twice. The nematodes were left to settle in the centrifuge tubes for 10 min and then 200 μL of the solution was pipetted from the bottom of the tube and added to 30 mL M9 solution in a second 50 mL centrifuge tube. The washing of *C. elegans* N2 by leaving them to settle in the conical centrifuge tube and transfer of 200 μL into new solution was repeated at least three times, before *C. elegans* N2 density was established using a Nematode Counting Chamber (Chalex Corporation, Wallowa, OR, United States). Fresh M9 solution was added or removed to reach a total of 1100 to 1450 nematodes per mL.

For the laboratory trials, avirulent *B. anthracis* Sterne 34F2 expressing the pSW4-GFPmut1 plasmid was used as a surrogate for fully pathogenic *B. anthracis* (see Sastalla et al., 2009). GFP expression is driven by the pagA promoter which is highly expressed during vegetative growth. Spores from this strain trap GFP produced during vegetative growth into the body of the spore and are fluorescent for at least 14 days post sporulation (Sastalla et al., 2009). As such, spore stocks for relevant experiments in the current study were at least 2 weeks old.

Bacterial cultures were maintained as frozen spore stock or as chilled working stock. Spores were produced in triplicate using the quantitative three-step method described by Tomasino et al. (2008). For standard maintenance, bacteria were grown on TSA for a minimum of 18 h at 37 ± 1°C and for bacterial enumeration in soil during experiments either Luria Bertani (LB) agar (Fisher Scientific, United Kingdom) or both TSA and PLET agar (both Nanologix, United States) were used. In brief, individual bacterial colonies from a working stock slope were inoculated into 10 mL of nutrient broth and incubated for 24 ± 2 h at 37 ± 1°C in a shaking incubator. The following day, 500 μL of culture was inoculated onto nutrient agar plates containing 5 μg/mL manganese sulfate monohydrate and spread evenly across the plate. Spore cultures were then incubated for 12–14 days at 37 ± 1°C before bacterial spores were harvested by adding 10 mL of 2–5°C sterile distilled water to the agar plates. Sterile spreaders were used to remove growth from plates and the resulting suspension was pipetted into 50 mL conical centrifuge tubes before spores were washed three times in sterile distilled water by centrifugation at 5000 rpm for 10 min at room temperature and stored at 2–5°C. Spore starting concentration for experiments in solution was ∼1 × 10^6^ CFU mL^-1^ or a 10-fold dilution thereof, which was achieved by diluting the spore stock with an appropriate volume of sterile distilled water. For soil microcosm experiments, the spore concentration was ∼1 × 10^6^ CFU g^-1^ in soil (or a 10-fold dilution thereof). Due to the large amount of time and number of consumables required for the protocols experiments were not repeated; instead, a nested approach was used in which three different spore stocks with inoculates from the same master bacterial culture stock were produced for the three replicates within one treatment group (for each day) to ensure reproducibility within the experiment. For microcosm studies in Turkey, soil collected at animal burial sites and containing virulent *B. anthracis* was spiked with additional *B. anthracis* Sterne 34F2 spores to bring the level of bacteria in the soil up to ∼10^3^ CFU g^-1^ in Microcosm Experiment 3 (to ensure detectable levels of CFUs at least in the control treatment) and up to ∼10^6^ CFU g^-1^ in Microcosm Experiment 4 (to ensure comparability with Microcosm Experiment 5).

### Materials and Soil Collection

All reagents and chemicals were obtained from Fisher Scientific (Loughborough, United Kingdom) or Sigma–Aldrich (Dorset, United Kingdom) unless otherwise stated in the text. Soil was collected in a forest near Gwaelod-Y-Garth, Wales, United Kingdom (51° 32′ 19.91″N, 3° 16′ 9.55″W) for Microcosm Experiments 1, 2, and 5. Soil used for experiments with fully virulent *B. anthracis* was collected from field sites A, Turkey (Microcosm Experiment 3) and D (Microcosm Experiment 4; coordinates of Turkish field sites are not provided for security reasons). Both Turkish field sites are known burial sites of anthrax positive animals and usually contain ∼10^1^–10^3^ CFU g^-1^.

Soil was collected fresh for each experiment, meaning that soil moisture content differed between experiments. Moisture content was measured by drying a portion of soil for 24 h at 60°C and comparing the overall weight before and after the drying period. The Welsh soil was comprised of free draining acid loam over rock (pH = 5.5–6.0); in contrast the Turkish soil was taken from a region rich in calcium due to the presence of limestone, with a pH > 6.0 and which is subjected to an annual cycle of local flooding due to seasonal snow melt, factors which have all been linked to the long term survival of virulent *B. anthracis*^1^ (Hugh-Jones and Blackburn, 2009).

### *C. elegans* N2 Ingestion and Survival on *B. anthracis*

*Caenorhabditis elegans* N2 was added to GFP-expressing *B. anthracis* Sterne 34F2 lawns on NGM and the population was left to develop for 3–4 days. The nematodes were then harvested as described above, immobilized for visualization using CytoFix fixation buffer (BD Cat. No. 554655, San Jose, CA, United States) and visualized under an IN Cell Analyzer 2000 (General Electric, Marlborough, MA, United States) at 395 nm from 10 to 100× magnification.

Early observations of *C. elegans* N2 maintained on *B. anthracis* Sterne 34F2 indicated that populations developed better on their standard laboratory food source of *E. coli* OP50. This was further investigated experimentally by inoculating NGM with 50 μL *B. anthracis* Sterne 34F2 (*n* = 5) or *E. coli* OP50 (*n* = 5) overnight liquid culture. Plates were incubated overnight at 37 ± 1°C before five adult, hermaphroditic *C. elegans* N2 were added to the NGM. Plates were incubated at 37 ± 1°C for 9 days to allow the *C. elegans* N2 to develop, which were then recovered from NGM as described above. *C. elegans* N2 suspensions were adjusted to a total volume 50 mL with M9 solution. The suspension was injected into a Nematode Counting Chamber in 1 mL aliquots, and then frozen for 10 min at -20°C to immobilize the nematodes before counting.

### Experimental Procedures for Microcosm Experiments

**FIGURE 1:**
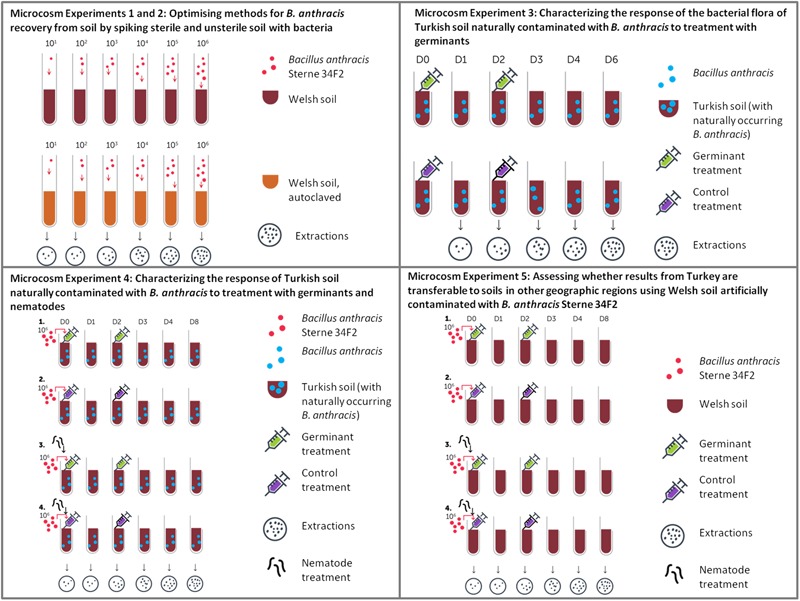
Microcosm experiments overview and aims.

An overview of soil microcosm experiments, their aims and how these build on each other is shown in **Figure 1**. Soil microcosms were prepared with 3 ± 0.1 g of non-sterile soil in 50 mL Nunc^®^ EZ Flip^TM^ conical centrifuge tubes. For Microcosm Experiment 1 (Recovery of *B. anthracis* Sterne 34F2 from autoclaved and artificially contaminated soil) and 2 (Recovery of *B. anthracis* Sterne 34F2 from artificially contaminated soil), soil samples were collected near at the Welsh field site with an overall moisture content of >90%.

Moisture content of site A soil used in Microcosm Experiment 3 (Effect of germinant treatment on cultivable soil bacteria in Turkish soil) was not determined as appropriate security measures to dry soil were not in place at the time of the experiment in the Turkish laboratories. Initial moisture content was 38.5% in site D soil (Microcosm Experiment 4: Effect of combined germinant and nematode treatment on *B. anthracis* spore numbers in contaminated soil from an animal burial site in Turkey) and 39.4% for Welsh soil (Microcosm Experiment 5: Effect of combined germinant and nematode treatment on *B. anthracis* Sterne 34F2 numbers in artificially contaminated soil from a Welsh field site).

At each time point (30 min after spiking for Microcosm Experiments 1 and 2; Days 1, 2, 3, 4, and 6 for Experiment 3; Days 0, 1, 2, 3, 4, and 8 for Experiments 4 and 5), all microcosms were extracted using the following method: 10 mL elution buffer (PBS, 28 mL L^-1^ Tween 80, 0.15 g L^-1^ L-Cysteine), formulated to promote spore recovery and minimize the carryover of antibacterial factors in the soil such as polyphenols (Beyer et al., 1995; BS EN 1276, 2009; Bishop, 2014; BS EN 13697, 2015), was added to each microcosm, which was then manually agitated until homogeneous, and two 1 mL aliquots were taken from each tube. One aliquot was used to determine the total viable cell count (non-heat-shock), and the other to determine the spore load (heat-shocked at 65°C for 1 h). Viable counts were obtained by 10-fold serial dilution in PBS and spread on LB agar plates (Fisher Scientific, United Kingdom; in duplicate, Microcosm Experiment 3) or TSA and PLET (in triplicate, Microcosm Experiments 1, 2, 4, and 5). LB and TSA plates were incubated overnight at 37°C, whereas PLET plates were incubated for two nights.

Observation and accurate recording of *B. anthracis* colonies for Microcosm Experiments 1, 2, 4, and 5 was simplified by adopting two types of ‘morphology controls’: one for pure *B. anthracis* Sterne 34F2 and a second for the endogenous soil microflora (ESMF). Both morphology controls were prepared on both PLET and TSA. The first was a streak plate of pure *B. anthracis* Sterne 34F2; the second was a series of 10-fold dilutions from the soil solution of non-spiked soil microcosms (undiluted to 10^-4^). These morphology controls provided a reference for visual identification of *B. anthracis* Sterne 34F2 colonies amongst ESMF, although such an approach will of course not completely negate the possibility of counting false positives particularly at the lower levels of detection when the ratio of *B. anthracis* to ESMF favors the latter and the ESMF presents a strong confounder (see also **Tables 1, 2**).

**Table 1:** Recovery of *Bacillus anthracis* Sterne 34F2 from sterile soil microcosms using tryptic soy agar (TSA) or polumixin B, lysozyme, EDTA and thallous acid (PLET) agar as the culture media (CFU g^-1^ soil).

Inoculum (spores g^-1^)	TSA						PLET					
	Total			Spore			Total			Spore		
	Mean	*SD*	% Rec	Mean	*SD*	% Rec	Mean	*SD*	% Rec	Mean	*SD*	% Rec
10^1^	2.08 × 10^1^	3.05 × 10^1^	208.33	0	0	0.00	7.41 × 10^1^	2.22 × 10^1^	74.07	0	0	0.00
10^2^	6.11 × 10^1^	4.91 × 10^1^	61.11	4.81 × 10^1^	4.12 × 10^1^	48.15	6.67 × 10^1^	5.27 × 10^1^	66.67	1.48 × 10^1^	2.42 × 10^1^	14.81
10^3^	6.56 × 10^2^	1.43 × 10^2^	65.56	5.89 × 10^2^	1.46 × 10^2^	58.89	5.93 × 10^2^	2.38 × 10^2^	59.26	6.11 × 10^2^	2.00 × 10^2^	61.11
10^4^	6.19 × 10^3^	2.05 × 10^3^	61.85	7.43 × 10^3^	1.51 × 10^3^	74.29	4.26 × 10^3^	1.42 × 10^3^	42.59	5.22 × 10^3^	8.98 × 10^2^	52.22
10^5^	7.37 × 10^4^	1.98 × 10^4^	73.70	6.22 × 10^4^	1.11 × 10^4^	62.22	6.33 × 10^4^	1.44 × 10^4^	63.33	4.59 × 10^4^	1.31 × 10^4^	45.93
10^6^	7.37 × 10^5^	1.51 × 10^5^	73.70	6.67 × 10^5^	1.92 × 10^5^	66.67	6.15 × 10^5^	1.76 × 10^5^	61.48	3.22 × 10^5^	1.24 × 10^5^	32.22

*The results are presented as log_*10*_ values. Error is represented as standard deviation (arithmetic mean total viable count and spore count ± SD; *n* = 3). % Rec, recovery percentage.*

**Table 2:** Recovery of *Bacillus anthracis* Sterne 34F2 from non-sterile soil microcosms using tryptic soy agar (TSA) or polumixin B, lysozyme, EDTA and thallous acid (PLET) agar as the culture media (CFU g^-1^ soil).

Inoculum (spores g^-1^)	TSA						PLET					
	Total			Spore			Total			Spore		
	Mean	*SD*	% Rec	Mean	*S*D	% Rec	Mean	*SD*	% Rec	Mean	*SD*	% Rec
10^1^	2.59 × 10^1^	3.64 × 10^1^	259.26	4.00 × 10^2^	6.08 × 10^2^	4000.00	0	0	0.00	6.00 × 10^2^	8.74 × 10^2^	6000.00
10^2^	1.85 × 10^1^	2.94 × 10^1^	18.52	1.11 × 10^1^	2.36 × 10^1^	11.11	1.11 × 10^1^	1.67 × 10^1^	11.11	1.11 × 10^1^	2.36 × 10^1^	11.11
10^3^	2.56 × 10^2^	4.18 × 10^2^	25.56	2.59 × 10^1^	5.72 × 10^1^	2.59	1.74 × 10^2^	6.83 × 10^1^	17.41	2.93 × 10^2^	1.54 × 10^2^	29.26
10^4^	4.48 × 10^3^	4.32 × 10^3^	44.81	4.44 × 10^3^	1.44 × 10^3^	44.44	2.28 × 10^3^	1.77 × 10^3^	22.78	4.15 × 10^3^	1.65 × 10^3^	41.48
10^5^	6.56 × 10^4^	1.70 × 10^4^	65.56	5.81 × 10^4^	1.63 × 10^4^	58.15	3.15 × 10^4^	1.30 × 10^4^	31.48	3.11 × 10^4^	1.43 × 10^4^	31.11
10^6^	6.26 × 10^5^	7.41 × 10^4^	62.59	6.22 × 10^5^	1.83 × 10^5^	62.22	3.26 × 10^5^	1.71 × 10^5^	32.59	3.78 × 10^5^	9.57 × 10^4^	37.78

*The results are presented as log_*10*_ values. Error is represented as standard deviation (arithmetic mean total viable count and spore count ± SD; *n* = 3). % Rec, recovery percentage.*

### Microcosm Experiments 1 and 2: Recovery of *B. anthracis* Sterne 34F2 from Autoclaved and Non-autoclaved, Artificially Contaminated Soil

The soil samples were separated into two aliquots, one of which was sterilized by autoclaving for 2 h at 121°C (for Microcosm Experiment 1). Soil was then divided among the microcosms and spiked with 10-fold increases in spore concentrations (10^1^–10^6^ spores g^-1^) at 50 μL and manually shaken to disperse spores. The low spiking volumes of spore suspension was chosen due to high soil moisture content (>90%) after soil collection as otherwise the soil would have been completely saturated with water.

### Microcosm Experiment 3: Effect of Germinant Treatment on Cultivable Soil Bacteria in Turkish Soil

Germinants in a well-defined mixture known to be highly effective for inducing germination (Ireland and Hanna, 2002) were added in a multi-step treatment at Day 0 (300 mM L-alanine, 15 mM inosine) and Day +2 (500 mM L-alanine, 25 mM inosine) to soil microcosms (∼10^2^–10^3^ naturally occurring *B. anthracis* spores g^-1^). The multi-step treatment aimed to keep germination sustained to ensure vegetative bacteria were available for nematode ingestion. A total volume of 2 mL liquid was introduced into each microcosm. Specifically, an extra 0.5 mL of *B. anthracis* Sterne 34F2 suspension (∼1 × 10^3^ CFU mL^-1^) was added to each microcosm and mixed to disperse spores evenly throughout the matrix to ensure a detectable level of bacteria at least in the control treatment. Either germinant (1 mL) or PBS (of variable volume depending on how much moisture had already been added with the germinant or spore suspension) was then added (Day 0) and the microcosms were mixed. At Day +2, an additional 100 μL of PBS (control) or germinant was added. Soil microcosms were in sealed 50 mL conical tubes to prevent evaporation, and stored at ambient temperature (22.0 ± 2.0°C).

### Microcosm Experiments 4 and 5: Effect of Combined Germinant and Nematode Treatment on *B. anthracis* Spore Numbers in Contaminated Soil from an Animal Burial Site in Turkey and Effect of Combined Germinant and Nematode Treatment on *B. anthracis* Sterne 34F2 Numbers in Artificially Contaminated Soil from a Welsh Field Site

Microcosms were spiked with *B. anthracis* Sterne 34F2 at approximately 1 × 10^6^ spores g^-1^ (Microcosm Experiment 4 – Site D soil: ∼3.5 × 10^5^ CFU g^-1^ naturally occurring virulent *B. anthracis* spores which was unusually high compared to other sampling years and other known contaminated *B. anthracis* spores sites) and germinant solution was added in a multi-step treatment at Day 0 (300 mM L-alanine, 15 mM inosine) and Day +2 (500 mM L-alanine, 25 mM inosine). A heterogeneous nematode population consisting of all age ranges of *C. elegans* N2 suspended in either the germinant solution or PBS on Day 0 was added at a total volume of 1100–1400 nematodes mL^-1^. A total volume of 600 μL liquid was added to each microcosm. Specifically, 100 μL of spore suspension (∼3 × 10^7^ CFU mL^-1^) was added to each microcosm and mixed to disperse spores throughout the matrix. Either 330 μL of PBS (control), germinant, or germinant-nematode suspension was then added (Day 0) and the microcosms mixed. At Day +2, an additional 170 μL of PBS (control) or germinant was added. Soil microcosms were sealed in conical tubes to prevent evaporation, and stored at ambient temperature (Turkish laboratory: 22.0 ± 2.0°C; Welsh laboratory: 20.8 ± 1.6°C).

### Statistical Methods

Data of viable counts for Microcosm Experiments 3, 4, and 5 were log_10_ (+1) transformed and analyzed as dependent variables using generalized linear models (GLM). For Microcosm Experiments 3 and 5, model fit was best with a Gaussian family and identity link function. For Microcosm Experiment 4, a quasipoisson family and an identity link function were used. Due to a higher number of native microorganisms in Turkish soil, the detection limit for *B. anthracis* was at 10^5^ CFU g^-1^ soil and highly variable, as opposed to a conservative 10^2^ CFU g^-1^ in Welsh soil. The higher limit of detection resulted in more noise in the data set, which could not be normalized using standard approaches such as a further transformation of data and adjustments with error and link functions within the GLM. Because results equal to zero were likely to be artifacts (i.e., we could not identify *B. anthracis* below the lower limit of detection due to high ESMF), these data points were removed for statistical analysis of the results of Microcosm Experiments 3 and 5, resulting in completely normally distributed data.

For GLM on cultivable microorganisms on Turkish soil (Microcosm Experiment 3), treatment (control, and germinants only), day and viable count type (total or spore viable count) were used as independent variables with interactions terms between treatment: day, treatment: viable count type and day: viable count type. For both GLMs on Turkish and Welsh decontamination datasets (Microcosm Experiments 4 and 5), treatment (control, germinants only, nematodes only, and a combination of germinants and nematodes), day, viable count type (total or spore viable count), and media (PLET, TSA) were treated as independent variables with interactions terms as in Experiment 3. A step-wise model reduction process was applied and standardized residuals from each model were first checked visually for normality and homogeneity of variance using a histogram, Q–Q plots, and fitted values. All analyses were performed in R 3.1.1 statistical package (R Development Core Team, 2014).

## Results

The aim of this study was to assess whether the application of germinants, alone or in combination with nematodes, could lead to a reduction of *B. anthracis* spore numbers in contaminated soil.

### Survival of *C. elegans* N2 Following Ingestion of *B. anthracis*

**FIGURE 2:**
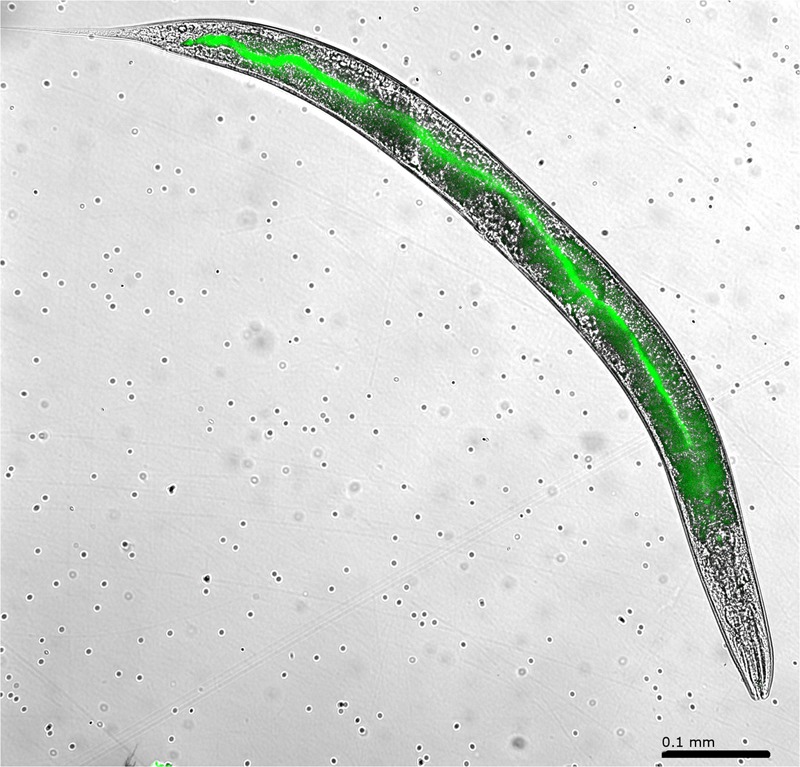
High resolution image (10× magnification) of *Caenorhabditis elegans* fed with green fluorescent protein (GFP)-*Bacillus anthracis* Sterne 34F2: the fluorescence clearly indicates that the nematode ingests the bacteria.

**FIGURE 3:**
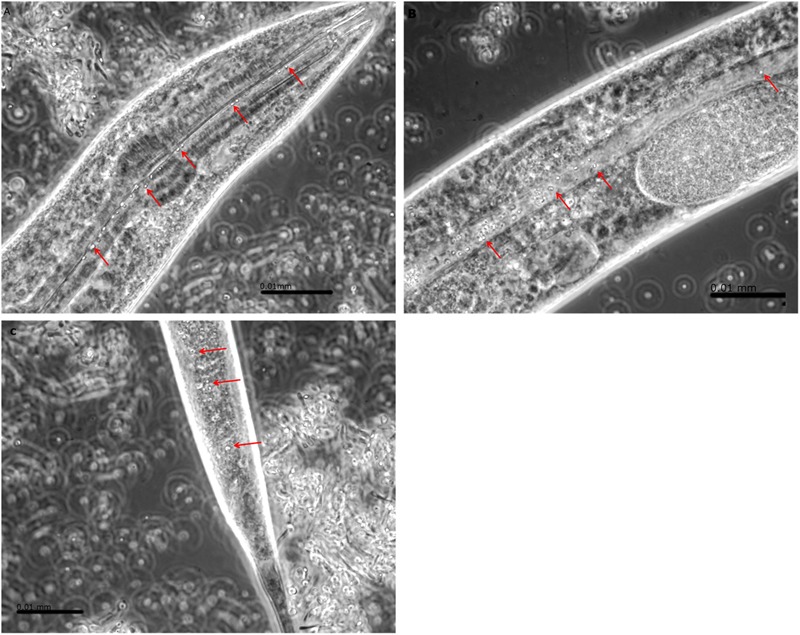
*Bacillus anthracis* Sterne 34F2 spores (selectively highlighted with red arrows) are clearly outlined in the pharynx **(A)**, gut **(B)**, and anus **(C)** of *Caenorhabditis elegans* N2 indicating that spores do survive ingestion by the nematode. Vegetative cells would be recognized by a more elongated shape typical of rod-shaped bacteria of the *Bacillus* spp. group.

**FIGURE 4:**
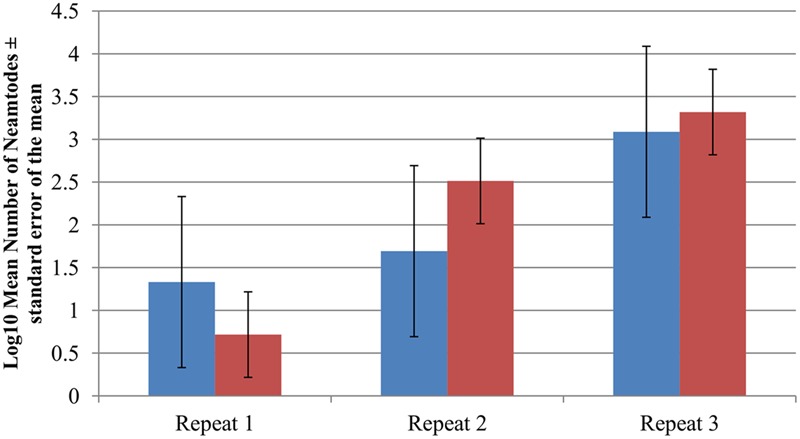
Log_10_ *Caenorhabditis elegans* N2 populations after 9 days of incubation while utilizing *Escherichia coli* OP50 (blue) or *Bacillus anthracis* Sterne 34F2 (red) as food source during three separate experimental repeats. *N* = 5 for each bacterial food source at each time point. Error bars represent the standard error of the mean.

*Caenorhabditis elegans* N2 cultures provided with GFP-tagged *B. anthracis* Sterne 34F2 clearly show that the nematodes ingest both vegetative bacteria and spores (**Figures 2, 3**). The images indicate that while spores pass through the gut intact, the vegetative bacteria are ingested confirming our initial hypothesis that *C. elegans* N2 can digest and neutralize the actively growing bacterium, but not the spore form of *B. anthracis*. Nematodes were visually observed to become lethargic and bloated after feeding on *B. anthracis* for an extended period. However, in terms of population development, no long-term negative effects were observed among nematodes that ingested the human pathogen: the nematodes survived on *B. anthracis* Sterne 34F2 cultures equally well as those feed *E. coli* OP50, their standard laboratory food source (**Figure 4**). Therefore, to decontaminate *B. anthracis*-contaminated soil, the use of nematodes is feasible if the bacteria are present in their vegetative form and not as a spore.

### Microcosm Experiment 1: Recovery of *B. anthracis* Sterne 34F2 from Autoclaved and Artificially Contaminated Soil

First, the recovery of *B. anthracis* Sterne 34F2 from autoclaved soil microcosms using selective (PLET) and non-selective (TSA) media was assessed. The lower limit of detection was 40 to 50 colony forming units (CFU) g^-1^ (**Table 1**); below this level, experimental variation caused results to be inconsistent. Although the number of colonies on PLET agar were slightly lower (40) than those seen using TSA (50), they were still within the same logarithmic range, suggesting that both media are equally effective for recovering *B. anthracis* from autoclaved soil.

### Microcosm Experiment 2: Recovery of *B. anthracis* from Artificially Contaminated Soil

Following the recovery of *B. anthracis* Sterne 34F2 from autoclaved soil, the effect of the ESMF on the ability to recover *B. anthracis* Sterne 34F2 was determined. Various concentrations of *B. anthracis* Sterne 34F2 were added to the non-sterile soil collected from Garth Mountain, southeast Wales, to test recovery success. *B. anthracis* Sterne 34F2 colonies were detected on TSA in samples taken from microcosms spiked with 10^1^ *B. anthracis* spores g^-1^ soil (**Table 2**); however, for an accurate determination of CFU g^-1^ soil in non-sterile microcosms, a minimum of 10^4^ CFU g^-1^ soil is desirable to ensure repeatability of results. The higher starting load has the advantage of reducing the overall background of ESMF from sample cultures (**Table 2**). Hence, to determine whether a combined germinant and nematode treatment could effectively reduce the *B. anthracis* spore load in contaminated soil, it was essential to work with detectable levels of *B. anthracis* in further trials to ensure repeatability across experiments.

### Microcosm Experiment 3: Effect of Germinant Treatment on Cultivable Soil Bacteria in Naturally *B. anthracis* Contaminated Turkish Soil

**FIGURE 5:**
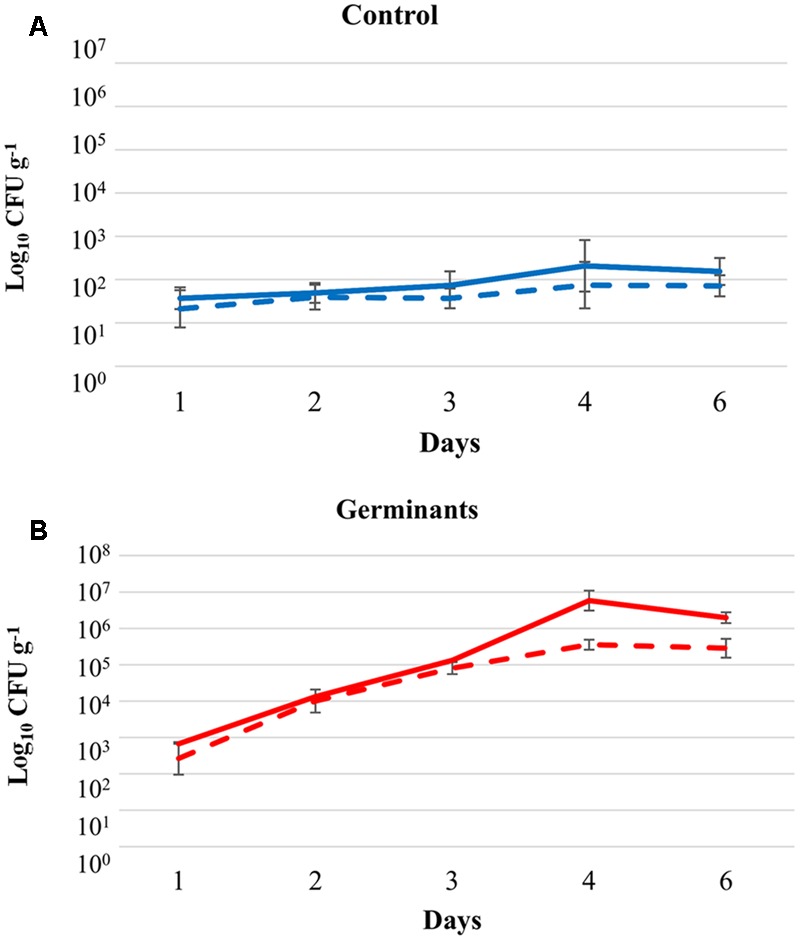
Kinetics of cultivable endogenous soil microflora on Luria Bertani agar in Turkish soil from a naturally contaminated animal burial site following treatment with **(A)** sterile phosphate buffered saline (blue) and **(B)** germinants (red). Microcosms were treated with germinants on Day 0 (300 mM L-alanine, 15 mM inosine) and Day +2 (500 mM L-alanine, 25 mM inosine Solid lines represent the viable count and dashed lines indicate spore counts ± SD (*n* = 3).

Our initial decontamination experiment assessed the impact of treatment with L-alanine and inosine as germinants on the total number of viable bacteria recovered from Turkish soil naturally contaminated with low levels of *B. anthracis* (∼10^3^ *B. anthracis* spores g^-1^). Exposure to the L-alanine and inosine germinant mixture on Day 0 and Day 2 of the experiment resulted in a 3.5 log increase in the soil microflora cultivable on Luria Bertani (LB) agar over the time course of the experiment (**Figure 5B**). In contrast, only a 0.5 log increase was observed after treatment with phosphate buffered saline (PBS) solution (**Figure 5A**; Generalized Linear Model [GLM from hereon]: total/spore recovery^∗^treatment interaction: *F*_1,212_ = 11.00, *P* = 0.001; GLM: treatment^∗^day interaction: *F*_4,212_ = 171.89, *P* < 0.001; GLM: total/spore recovery^∗^day interaction: *F*_4,212_ = 10.15, *P* < 0.001). Unfortunately, the low levels of *B. anthracis* relative to the ESMF in the soil meant that the increase of the target bacterium due to the germination and replication of the pathogen could not be determined.

### Microcosm Experiment 4: Effect of Combined Germinant and Nematode Treatment with Spore Numbers in Contaminated Soil from an Turkish Animal Burial Site

**FIGURE 6:**
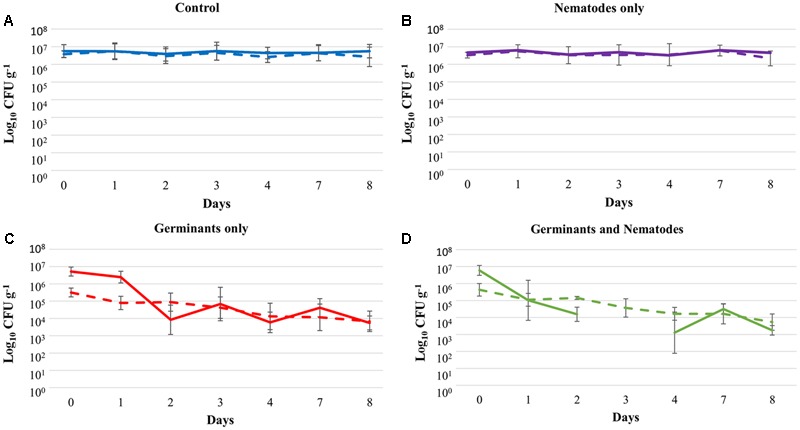
Kinetics of *Bacillus anthracis* recovery from Turkish soil supplemented with spores of the *B. anthracis* Sterne 34F2 strain (final spore concentration ∼10^6^ CFU g^-1^) after treatment with **(A)** sterile phosphate buffered saline, **(B)** nematodes (*Caenorhabditis elegans* N2), **(C)** germinant, and **(D)** germinant and nematodes (*C. elegans* N2). Microcosms were treated with germinant on Day 0 (300 mM L-alanine, 15 mM inosine) and Day +2 (500 mM L-alanine, 25 mM inosine). Microcosms were treated with nematodes on Day 0 only. Solid lines represent the total viable count and dashed lines indicate spore counts ± SD (*n* = 3). Total viable counts on Day 3 for the combined germinant and nematode treatment could not be determined because of high levels of endogenous soil microflora.

Due to the low numbers of *B. anthracis* spores present in contaminated soil from the animal burial site (∼3.5 × 10^5^ CFU g^-1^), we artificially increased the *B. anthracis* spore load to a final concentration of ∼10^6^ CFU g^-1^. As expected, the number of bacteria in soil treated with PBS did not change significantly over the course of the study (**Figure 6A**). In contrast, soil treated with germinants demonstrated 0.6 log germination by Day 1 and a 3 log reduction in total viable count by the end of the trial (**Figure 6C**). These results suggest that, rather than replicating like the rest of the ESMF (**Figure 6C**), *B. anthracis* numbers actually declined when treated with germinants.

To determine if we could further reduce the number of viable *B. anthracis* in artificially contaminated soil, separate microcosms were treated with a combination of germinants and *C. elegans* N2. While treatment with nematodes alone had no effect on *B. anthracis* numbers (**Figure 6B**) a combination of germinants and nematodes led to a 3.5 log reduction by Day 8 which differed significantly from other treatments (**Figure 6D**; GLM: treatment^∗^day interaction: *F*_18,298_ = 15.16, *P* < 0.001). Total and spore recovery from microcosms differed also across days (**Figures 6C,D**; GLM: total/spore recovery^∗^day interaction: *F*_6,263_ = 4.85, *P* < 0.001). There was no significant difference in recovery between TSA and PLET agar types (GLM: *P* > 0.05). For the germinant and nematode treatment, viable counts on Day 3 could not be determined because of high levels of ESMF.

### Microcosm Experiment 5: Effect of Combined Germinant and Nematode Treatment on *B. anthracis* Sterne 34F2 Numbers in Artificially Contaminated Soil from a Welsh Field Site

**FIGURE 7:**
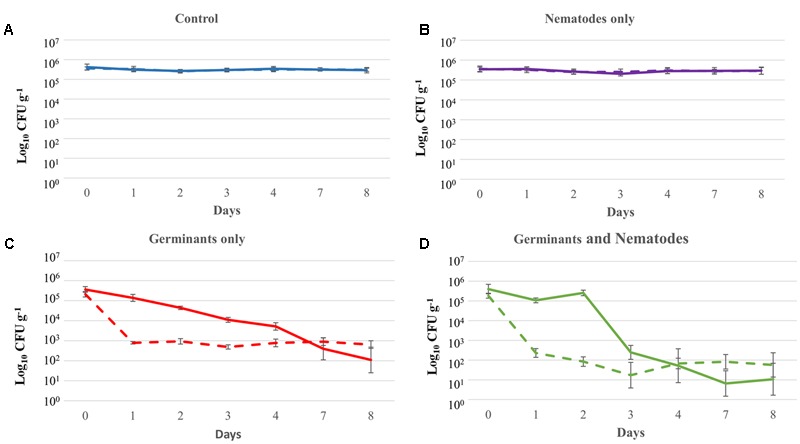
Kinetics of *Bacillus anthracis* Sterne 34F2 recovery from spiked Welsh soil (final concentration ∼10^6^ CFU g^-1^ spores) following treatment with **(A)** sterile phosphate buffered saline, **(B)** nematodes (*Caenorhabditis elegans* N2), **(C)** germinant, and **(D)** germinant and nematodes (*C. elegans* N2). Microcosms were treated with germinants on Day 0 (300 mM L-alanine, 15 mM inosine) and Day +2 (500 mM L-alanine, 25 mM inosine) and with nematodes on Day 0 only. Solid lines represent the total viable count and dashed lines represent spore counts ± SD (*n* = 3).

To determine if soil composition had an effect on the efficiency of germination and the ability of the nematodes to consume their food source, we repeated the same experiment as in Microcosm Experiment 4 using soil collected from a site on Garth Mountain in southeast Wales that had no recorded history of *B. anthracis* spore contamination. The results in this soil (**Figure 7**) were similar to results seen in the Turkish soil (**Figure 6**).

Spore numbers significantly decreased with application of L-alanine and inosine plus nematodes. While treatment with nematodes alone had no effect on *B. anthracis* numbers (**Figure 7B**), a 4.6 log reduction was observed when nematodes were used in combination with germinants (**Figure 7D**) which was significantly different from that achieved with germinants alone over the duration of the experiment (3.5 log; GLM: treatment^∗^day interaction: *F*_18,298_ = 15.16, *P* < 0.001). The total number of viable bacteria and spores recovered was dependent on treatment and differed over time (**Figure 7**; GLM: total/spore recovery^∗^day interaction: *F*_6,298_ = 27.51, *P* < 0.001; GLM: total/spore recovery^∗^treatment interaction: *F*_3,298_ = 6.94, *P* < 0.001).

During the last few days of the experiments for both Welsh and Turkish soil, enumeration of *B. anthracis* colonies was considerably more challenging from microcosms which had been treated with germinants. As *B. anthracis* colonies declined and the limit of detection was reached, the number of ESFM colonies stayed the same or increased (consistent with results from Microcosm Experiment 3). Particularly, samples that were not autoclaved to determine the overall *B. anthracis* colonies had to be diluted further than autoclaved samples (for spore enumeration) so increasing the error in the collected data and increasing the likelihood of false negatives. The different agar types (PLET and TSA) did not affect recovery (GLM: *P* > 0.05).

## Discussion

Despite a large focus of research on the decontamination of *B. anthracis* in the last 10 years, (Calfee et al., 2011; Luu et al., 2011; Buhr et al., 2012), studies into complex matrices such as soil are only just emerging (e.g., Omotade et al., 2014). This research has shown that compounds utilized in the decontamination of *B. anthracis* in a sterile environment may also be suitable in the field (EPA, 2013; Bishop, 2014; Celebi et al., 2016). Here we show that germinants by themselves reduce overall *B. anthracis* spore count significantly in two different soil types which combined with results from Bishop (2014) indicate that germinants alone can be effective decontaminants across various soils. Further, the added reduction in *B. anthracis* spore levels when *C. elegans* N2 are included in the treatment regimen could significantly reduce the spore load and mitigate the potential health threat (Raber et al., 2001, 2004; Canter, 2005).

Currently, the fate of bacteria after treatment with germinants is unclear, although several scenarios are possible. From the data presented in this study, nematode predation had a measurable impact on the total number of viable *B. anthracis* cells but only after the spores had been triggered to germinate. This observation is supported by visual microscopic examination (Choi and Richter, personal observations) and previous research on *C. elegans* utilizing *B. subtilis* as a food source (Laaberki and Dworkin, 2008a,b; Engelmann and Pujol, 2010). Further, the reduction of spores in soil through germinant treatment alone illustrates the effect that other factors may have on elimination of the bacteria. For instance, lack of nutrients may prevent the newly germinated bacterium from replicating and re-forming spores (Omotade et al., 2014). Other members of the soil microflora may compete with the newly emerged vegetative bacteria for nutrients and space or could suppress *B. anthracis* numbers through the production of antibiotic compounds (Klobutcher et al., 2006; Saile and Koehler, 2006; Rønn et al., 2012). Indeed, the increase in ESMF (**Figure 4**) in the soil after addition of germinants indicates that *B. anthracis* may find itself outcompeted when in an environment where its replication is limited (Saile and Koehler, 2006) instead of its standard mammalian host. The ESMF triggered to germinate by alanine and inosine clearly benefit from available nutrients and the favorable conditions in the laboratory, although it is likely that the bacterial population growth in soil would have eventually stopped with the reduction of nutrients in the soil. Further reasons for the reduction of *B. anthracis* in soil include possible predation of the bacteria by eukaryotic predators such as protozoa (Dey et al., 2012); and lastly, bacterial numbers may be reduced by the infection with lytic bacteriophages which are known to present in the soil (Gillis and Mahillon, 2014).

It is expected that *C. elegans* N2 will ingest bacteria within the soil indiscriminately as its preferred habitat are rotten fruit and the food sources available there (Stiernagle, 2006; Neher, 2010); hence, a high initial level of nematodes during any remediation efforts would be essential and further research on the minimum number of nematodes needed is advisable. Although treatment with *C. elegans* N2 is likely to be effective in reducing overall *B. anthracis* levels in soil, it is not a soil nematode *per se* and in the wild is found mainly in compost or other rotten material (Felix and Duveau, 2012). Thus it is unlikely to be able to survive in the soil long term and only in the initial phase where ESMF is high due to the addition of germinants, maintaining the ecosystem balance. Repeated treatments may be required to completely eliminate the pathogen with treatment cycles spaced so that it coincides with nematode die off in the soil.

If further research on the long term survival of *C. elegans* N2 in soil finds that ecosystem perturbation is a concern, due to, for instance, *C. elegans* N2 reverting to the dauer larval stage once environmental conditions become unfavorable, the isolation of indigenous nematodes is a feasible alternative. The use of locally isolated nematodes may have the advantage of preventing translocation of exotic species and makes use of local co-adapted predator-prey dynamics; however, isolation and enrichment of cultures can take several weeks and is dependent on environmental factors (Schelkle, personal observations).

Finally, despite previous studies indicating an advantage of using PLET agar over TSA (Sjøstedt et al., 1997; Dragon and Rennie, 2001), the use of both agar types during the current study indicated no difference in the efficiency of the recovery of *B. anthracis*. Hence, for large scale studies such as the current one, TSA can be a feasible alternative for the more expensive PLET medium which also requires chilling for storage and is variable in efficacy. Further, conclusive identification of colony forming units recovered from unsterile soil remains a challenge without confirmation using reliable DNA tests which could not be deployed in our study (and indeed would not distinguish between vegetative cells and spores). Nonetheless, the use of appropriate statistical methods that allow to account for high variability in recovered numbers (particularly at the lower limits of detection where the ESMF is a strong confounder) provides a good indication of whether treatments are successful or not.

## Conclusion

A combined germinant-nematode approach has clear benefits for remediation of *B. anthracis* contaminated soil. However, in acknowledgement of (a) the limit of detection experienced in the current study and (b) the lack of knowledge on a safe level of *B. anthracis* spores in the environment (Raber et al., 2001, 2004; Canter, 2005), it is strongly recommended to follow up any seemingly successful decontamination effort with standard molecular methods to confirm remediation success (Beyer et al., 1995; Sjøstedt et al., 1997; Ryu et al., 2003; Vahedi et al., 2009; Fasanella et al., 2013; OIE, 2013). Due to the nested experimental approach, it may be beneficial to repeat the experiments with additional soil types. Once completed, a combined germinant-nematode approach has clear benefits, however, full-scale field trials to confirm the results of the current study are needed. Further, additional research is necessary to establish the effect of environmental conditions such as changing temperature or drainage of germinants on the overall efficacy of the combined germinant and nematode treatment.

## Author Contributions

We can confirm that BS, YC, LB, and TG contributed to the conception and design of the experiments. BS, YC, WR, FB, EC, MW, and MS contributed to the acquisition, analysis and interpretation of the data. BS, YC, LB, and TG contributed to the drafting of the article. All authors participated in the critical review of the draft paper.

## Conflict of Interest Statement

The authors declare that the research was conducted in the absence of any commercial or financial relationships that could be construed as a potential conflict of interest.

## Footnotes

1 http://www.landis.org.uk/soilscapes/
