# Supplementary material for: Caenorhabditis elegans Predation on Bacillus anthracis: Decontamination of Spore Contaminated Soil with Germinants and Nematodes
Source: Front Microbiol. 2018 Jan 5;8:2601. doi: 10.3389/fmicb.2017.02601 (PMC5770795; doi:10.3389/fmicb.2017.02601)
Supplement: Supplementary file 1 [file Data_Sheet_1.DOCX]

**Turkish National Regulations governing the work of the Bio-Safety Level-3 laboratories at Kafkas University, Turkey**

1. “Biyolojik etkenlere maruziyet risklerinin önlenmesi hakkında yönetmelikˮ 10 Haziran 2004 RG; Sayı 25488.  Bu yönetmelik 18/9/2000 tarih ve 2000/54/EC sayılı Avrupa Birliği (AB) Konsey Direktifinin Uyumlulaştırılması  kapsamında yayımlanmıştır. (Regulation on the prevention of the risk of exposure to biological agents).

2. ‟Enfeksiyöz Madde ile Enfeksiyöz Tanı ve Klinik Örneği Taşıma Yönetmeliği” 25 Eylül 2010 RG; Sayı 27710.  Bu yönetmelik 30/11/2005 tarih ve 5434 sayılı Kanun ile kabul edilen Tehlikeli Malların Karayolu ile Uluslararası Taşımacılığına ilişkin Avrupa Antlaşmasına paralel olarak hazırlanmıştır. İyi çalışmalar. (Infectious substances and infectious diagnosis and clinical sample Transport Regulations 2010).

3.Veteriner Biyolojik Ürünlerin depolanmasi Tasinmasi Yön. (Directional transport of storage of Veterinary Biological Products).

4. Tibbi Atiklarin Kontolü Yönetmeligi. (Regulation of medical waste management and control).

5. Tibbi Lab Yön Basbakanlik Mevzuati Gelistirme ve Yayin Genel Müdürlügü. (Prime Ministry General Directorate of Legislation Development and Publication of Medical Lab Course).

6. Veteriner Biyolojik Ürünlerin  Ilgili Yönemelik 2013. (Regulations concerning the Veterinary Biological Products 2013).
